# A Singular Case of Malformation

**Published:** 1867-07

**Authors:** J. M. Johnson

**Affiliations:** Atlanta, Ga.


### ARTICLE II.

*A Singular Case of Malformation.* By J. M. Johnson, M. D., of Atlanta, Ga.

On the 2d day of June, 1867, I was called to visit the infant son of a most respectable gentleman of this city. The child was three days old, having been born on the 30th day of May. The meconium had not passed away; castor oil had been administered once, and after the lapse of a short time ejected. The vomiting continued at intervals: the matter thrown up was of a stercoraceous character.

This had been the case for twelve hours before I was called to see the patient. It had passed a drop or two of urine on the second day; and at my first visit on the third day, and whilst examining it, a few drops more escaped. I ordered a warm bath and an enema: both were administered. In an hour the father came with the information that the fluid used as an injective could not be made to ascend the bowel, and without the least volition on the part of the child, returned.

I went again, and upon introducing a bogie, found that it only passed three-fourths of an inch. I then introduced the little finger of my left hand, and found the rectum closed. Upon examining the abdomen I found a tumor as large as a hen's egg, over the region of the segmoid flexure, attended with considerable discoloration of the skin, and some hardness. I requested Dr. W. F. Westmoreland, Prof. Surgery in Atlanta Medical College, to see the patient with me. He made a most searching examination, the result of which was that there was no rectum: even the rudimental elements were wanting. The bladder could be felt easily enough, and as it lay in the track where the rectum should have been found, was at first mistaken for this gut. It was clearly demonstrated that there was an absence of rectum with concervation of anus, and consequently two cul-de-sacs, one of the anus, and the other of the colon at the segmoid flexure. Let it be borne in mind, that the bladder could not be felt above and under the symphysis, notwithstanding three days had elapsed without the passage of more than a teaspoonfull of urine. The pelvic basin seemed to be free from any unnatural fullness, such as would have been the case if the bladder had held its normal place, and contained the accumulations of three days.

After this most mature and thorough investigation of the case, we declined to operate at that time. The diagnosis was anal cul-de-sac, with absence of rectum; a tumor in the left iliac, caused by the accumulated feces, and most probably a cul-de-sac of the colon, presenting the apperance of inflammation; the vomiting continued, of green matter; the extremities cold; no pulse at the wrist. To operate we must pass a trocar from the anus to the tumor, a distance of more than three inches, with the bladder in the track, and liable to be wounded; but this danger passed, and the sac reached, could we expect to keep the opening of the distended bowel in contact with that made by the trocar? and if we could, would not inflammation be excited by the presence and extravasation of the feces in their decent to find an outlet? and, finally, would not the inflammation thus aroused close up the opening? Such a multitude of difficulties induced us to dismiss all idea of an operation. Gross, Nelaton, and Petit, all discourage it, and there is not an instance of a successfull termination on record of such cases.

On the 5th day two circumstances occurred: the tumor over the segnoid disappeared, and the child passed urine freely, and continued to do so up to the tenth day, when it died.

Some delay occurred in making post-mortem, on account of the absence of Dr. Westmoreland.

I procured the assistance of Dr G. G. Crawford, and Dr. R. C. Word. At the request of both, Dr. Word and Myself, Dr. Crawford took the knife. Incisions were made on each side of the genitals, dividing the urethra in the middle; the parts were hooked up out of the way; pubis divided; anus and sphincters dissected, disclosing cul-de-sac as above stated, three-fourths of an inch deep; half an inch above was found the rounded end of the colon much distended, with meconium and gasses, and the fundus of the bladder attached by cellular membrane to it. The conviction is fixed in my mind *that the bladder occupied the track of the rectum*, and by the decent of the colon into the pelvis, the the bladder was restored to its normal position, and the urine was passed without difficulty after the fifth day. It will be remembered, that on the fifth day the tumor disappeared from the left flank, and pari-passu with this; the urine was freely discharged; the child also became calm, as if nothing was the matter, and remained so for two days. This theory is the more plausible, in view of the fact, that the track of the rectum was sufficiently open to admit the colon, swelled to double its size, and in view of the further fact that no abnormal growth resembling horn; no cord resembling the umbillical cord, as discribed by authors, with celular membrane extending in all directions, were found. In malformations of this kind, it is not an unfrequent occurrence, as we will presently show, to find an opening from the gut into the bladder, urethra, and uterus. Compensation is a law of nature, and although there was no opening into the bladder, it is plain that this organ occupied the place allotted by nature to the rectum, or some other economic disposition would have been made of the cavity, which was not the case.

Boyer says this is an abnormal conformation, and the aid of art can save but a small number.

Petit found a case according to this author, where the sphincters were perfect, and an occlusion occurred in the rectum above the sphincters. He used the pharangetome, and had no difficulty in making the incision—the membrane offering little resistance, and the meconium was discharged. The child lived two months. This operation was made two days after birth. This operation to be successful, he continues, should be made early.

Sometimes there is so great a space between the deficient end of the rectum and the sac containing the meconium, that it would be difficult to reach, even when you had made the proper diagnosis. The opening is rarely large enough, and the feces are liable to re-accumulate, and their retention cause the death of the child at last. (Memoir's Academy of Surgery, page 250.)

Engerron, (Ib.) page 253, gives the case of a child which, four days after birth vomited up everything, and could not pass the meconium, although the external anus was well formed. He introduced a stilet, but finding a strong hard body, was obliged to use instead of the stilet, a triangular needle, which gave issue to a great quantity of fecal matter: the excrements, however, accumulated again, and the child died at the end of a month. Autopsy disclosed at the end of the rectum a species of knot, resembling the umbillicus of an adult. Sometimes the obstacles are of such a nature as to prevent the success of an operation.

Trion gives the case of a female infant, of which the anus had a good conformation, but upon introducing the stilet, found resistance. The child died at the end of three days. Upon opening the cadaver, and the anus being dissected, it was found that above the length of the finger from the orifice, there was a membrane which opposed resistance. It was about ten lines in thickness, and about the consistence of horn. In cases like this, however well performed, the operation would not likely be successful. Usually, when the real difficulty is perceived, the opening is rarely made large enough, and he might have added, still more difficult to keep it so, owing to the presence of inflammation caused not only by the operation, but by constant contact of irritating matter.

Petit found a case of imperforate rectum, and an operation failed to reach the meconium. About three hours after, a dark sack protruded transversely over the wound, and which, when opened, discharged the meconium. It proved to be a hernia of the bowel driven down by the straining of the child; after this the child was easy, but died in seven or eight days.

The hernia was formed by the posterior part of the rectum. The portion of the rectum covered by the sphincters was entirely effaced, without a vestige or appearance of a cavity.

Another case of the same kind was first operated on with the lancet, and afterwards with the trocar; but the child died next day. (Petit.)

These operations, and a great many others like them, all show that there can be no success where the rectum is wanting, or where it is malformed, and it would be proper to abstain from it, if the absence of the rectum can be known. Authors cite a great many cases where the rectum terminates in a cul-de-sac at the base of the sacram, and even fails altogether, and asks, very properly, what is the use of an operation under such circumstances? (Petit.)

Speaking of the absence of rectum with concervation of anus, Nelaton says "in these cases there are two cul-de-sacs of greater or less depth, the cul-de-sac anal and the cul-de-sac rectal. Between these two cul-de-sacs is found not a new membrane as in simple imperforation, but an interval more or less considerable, filled by tissues of an aspect and consistence, variable, closing up at times a cord, hard and impenetrable, the last vestige of intestine. In some cases it is filled by cellular tissue altogether, that found in inter-organic spaces. (Petit.) A case is mentioned by Monsieur Jouvinal Delvin court, of a female child which had an anus well formed exteriorly, but closed at an inch high. Several incisions or punctures were made, without success. The child died, and autopsy demonstrated that the segmoid illiac terminated by a pocket at the point of the sacro-vertebral angle, and was fixed to the superior part of the sacram; the interval from the base of this pocket to the depression of the anus was supplied by cellular tissue, and by a fold (Sallie) of the vagina. Monsieur Bordinet has seen a case in which the anus is well formed, terminate at one centimetre. He deferred the operation until the third day; the little finger introduced into the anus, detected neither softness nor fluctuation. A pointed bistory was passed to the depth of one and a half centimetres, which did not open the rectum. Four days after, two new punctures, which penetrated three centimetres above the cul-de-sac, were made with a like result. Death occurred on the 20th day.

The autopsy showed the cul-de-sac anal profound to the depth of one centimetre. That it was attached by a cord to the rectum full one and a half centimetres in length, and the intestine terminated above the cord, by a cul-de-sac regularly rounded. The dilation was such, that this part of the intestine filled exactly all the cavity of the pelvis.

Our own great Author, Dr. Gross, speaking of the deformity we are now considering, says: "In rare cases, the absent canal is represented by a fibro-ligamentous cord attached to the colon, and ascending along the sacram towards the neck of the bladder, where it is lost in the cellular substance." Again, "an operation similar to the one just described, may be performed when the rectum is absent, although with hardly any possibility of a successful issue, for even supposing that the cause could be reached, the child would be likely to perish from peritoneal inflammation, induced either by the incision of the intestine, or by the extravasation of fical matter." Dr. Gross justifies the operation only on the ground that there is no other means of relief, although not an instance of cure can be found upon record.

Capt. McDaniel of this city, relates, as coming within his own knowledge, a very curious circumstance, which can be verified by a number of witnesses. He says that Mr. John E. Gadsey, of Franklin, Williamson County, Tennessee, eighteen miles South of Nashville, raised a male hog to be eighteen months old, with imperforate anus. The animal was always fat, and in every particular properly developed. After retaining his food for a certain length of time, feeding as other hogs, vomiting would ensue, and then with an appetite as keen as ever, go to his meals again. He was killed at the age of eighteen months, and to all appearances was in perfect health and thoroughly fat.

